# Outstanding compressive creep strength in Cr/Ir-codoped (Mo_0.85_Nb_0.15_)Si_2_ crystals with the unique cross-lamellar microstructure

**DOI:** 10.1038/s41598-017-04163-0

**Published:** 2017-06-21

**Authors:** Koji Hagihara, Takaaki Ikenishi, Haruka Araki, Takayoshi Nakano

**Affiliations:** 1 Department of Adaptive Machine Systems, Graduate School of Engineering, Osaka University, 2-1 Yamadaoka, Suita, Osaka, 565-0871 Japan; 2 Division of Materials and Manufacturing Science, Graduate School of Engineering, Osaka University, 2-1 Yamadaoka, Suita, Osaka, 565-0871 Japan

## Abstract

A (Mo_0.85_Nb_0.15_)Si_2_ crystal with an oriented, lamellar, C40/C11_b_ two-phase microstructure is a promising ultrahigh-temperature (UHT) structural material, but its low room-temperature fracture toughness and low high-temperature strength prevent its practical application. As a possibility to overcome these problems, we first found a development of unique “cross-lamellar microstructure”, by the cooping of Cr and Ir. The cross-lamellar microstructure consists of a rod-like C11_b_-phase grains that extend along a direction perpendicular to the lamellar interface in addition to the C40/C11_b_ fine lamellae. In this study, the effectiveness of the cross-lamellar microstructure for improving the high-temperature creep deformation property, being the most essential for UHT materials, was examined by using the oriented crystals. The creep rate significantly reduced along a loading orientation parallel to the lamellar interface. Furthermore, the degradation in creep strength for other loading orientation that is not parallel to the lamellar interface, which has been a serious problem up to now, was also suppressed. The results demonstrated that the simultaneous improvement of high-temperature creep strength and room temperature fracture toughness can be first accomplished by the development of unique cross-lamellar microstructure, which opens a potential avenue for the development of novel UHT materials as alternatives to existing Ni-based superalloys.

## Introduction

There is a strong demand for the development of a novel ultrahigh-temperature (UHT) structural material as alternatives to the Ni-based superalloys, which can withstand the use above 1400 °C without requiring cooling. Such materials would increase the thermal efficiency of combustion systems such as jet engines for airplanes, and next-generation ultra-high temperature gas turbine systems in power plants such as the gas turbine combined cycle (GTCC). Thus, the replacement of Ni-based superalloys to such UHT materials would drastically reduce the CO_2_ gas emission that causes global warming. Transition-metal disilicides are potential UHT structural materials due to their high melting temperatures, low densities etc^1–7^. For their practical application, we have proposed two-phase crystals in which different disilicides with properties that complement each other are combined. A combination of C11_b_ phase (such as MoSi_2_) and C40 phase (such as NbSi_2_) with considerable ductility and superior high-temperature strength by controlling the loading orientation, respectively, is considered the most promising candidate^8–26^. In Nakano *et al*.^11^, we fabricated a C40/C11_b_ two-phase crystal with an oriented lamellar microstructure. Through the heat treatment of the (Mo_0.85_Nb_0.15_)Si_2_ C40 single-phase single-crystals grown by the floating zone (FZ) method, C11_b_ phases with ~(Mo_0.94_Nb_0.06_)Si_2_ composition were precipitated in the ~(Mo_0.79_Nb_0.21_)Si_2_ C40 matrix phase. Notwithstanding the observed improvement in the mechanical properties of the silicide crystal due to the development of lamellar microstructure^15, 21, 24^, its room temperature fracture toughness and high-temperature creep strength were still insufficient for practical application. To further improve these properties, studies^22^ aimed at controlling the lamellar microstructure by a minute amount of alloying element additions have been conducted. In the research process, we found the novel development of the unique “cross-lamellar microstructure” in the Cr/Ir-codoped crystal, as the details are described later. Development of such microstructure was never reported until now, and it was found to result in a drastic increase in fracture toughness^26^. However, the variation in creep properties at high-temperatures around 1400 °C, which is more essential for realizing the practical application of this crystal as an UHT material, remains unknown. Based on these background, we have examined the high-temperature compressive deformation behavior of the CrIr-codoped (Mo_0.85_Nb_0.15_)Si_2_ crystals (CrIr-added crystal) in this study. Results of the current study were compared to previous study of non-added crystal^24^ and Cr-only-added and Ir-only-added crystals, and then the effectiveness for the improvement of the creep strength and its mechanism by the development of “cross-lamellar microstructure” were clarified.

## Results

**Figure 1:**
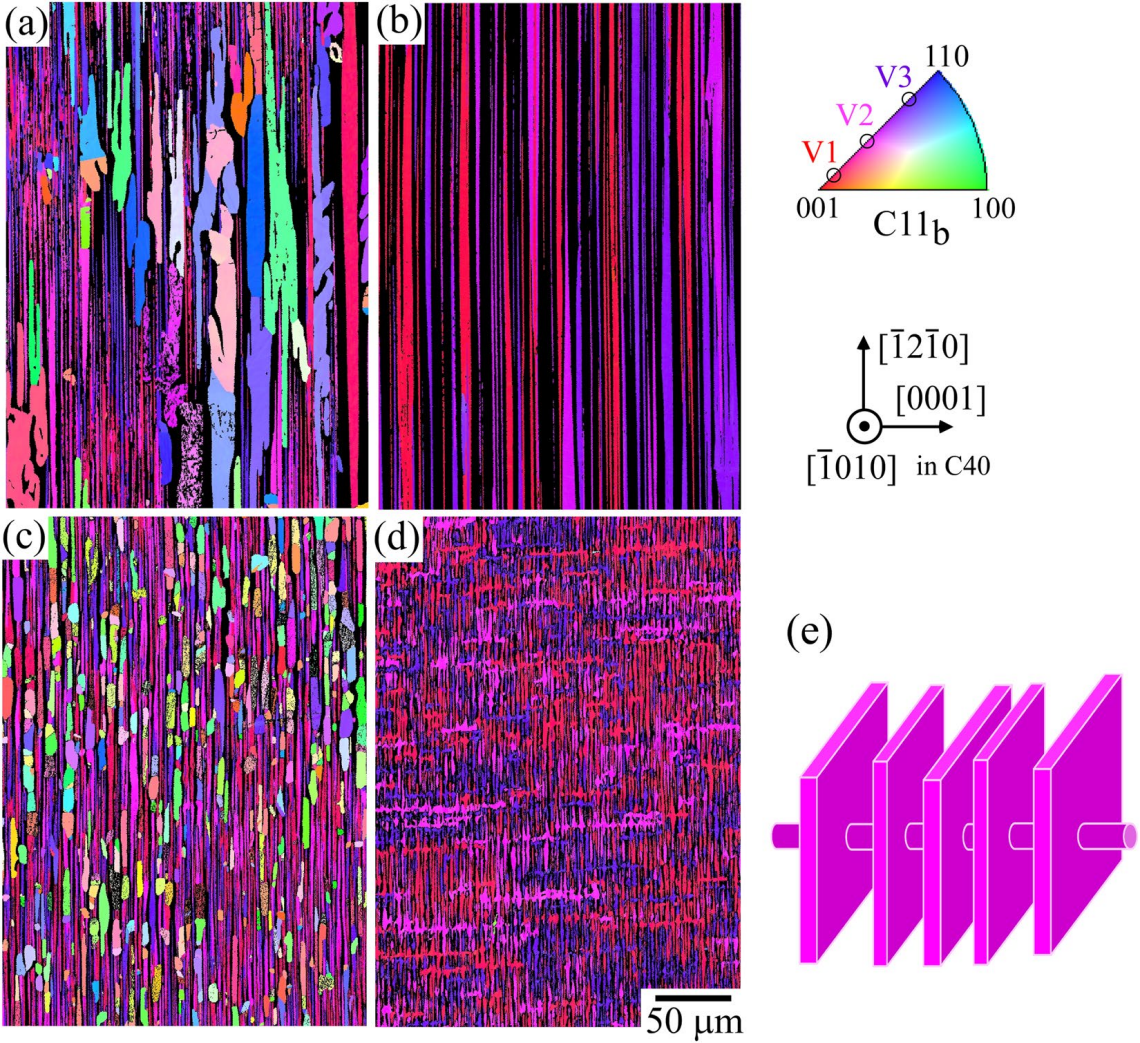
Microstructure variations depending on the added element. (**a**–**d**) Crystal orientation maps of the constituent C11_b_ phases in the FZ-grown crystals annealed at 1400 °C for 168 h examined by SEM-EBSD, according to the previous study^26^. (**a**) Nonadded (Mo_0.85_Nb_0.15_)Si_2_ ternary crystal, (**b**) 1 at.% Cr-added crystal, (**c**) 1 at.% Ir-added crystal, (**d**) 0.5 at.% Cr and 0.5 at.% Ir-coadded crystals. (**e**) Schematic of the shape of the C11_b_-phase grains formed the “cross-lamellar” morphology in the CrIr-added crystal. Noting that in Fig. 1(a–d) the crystal orientation map was analyzed along a direction rotated towards [11 $\overline{2}$ 0] by 15° from the observed [10 $\overline{1}$ 0] direction, to distinguish the orientation colors of the three variant C11_b_-phase grains.

Figure 1(a–d) describe the crystal orientation maps of the precipitated C11_b_ phases analyzed by the electron backscatter diffraction in a scanning electron microscope (SEM-EBSD) in the annealed two-phase crystals^26^. The black parts and the colored grains represent the C40-matrix-phase and precipitated C11_b_-phase grains, respectively. In addition, the optical microscope (OM) images of the specimens are shown in Supplementary Figure S1. The lamellar microstructure in the (Mo_0.85_Nb_0.15_)Si_2_ C40/C11_b_ two-phase crystal exhibits distinct crystallographic orientation relationships of (0001)_C40_//{110)_C11b_ and < $\bar{1}2\bar{1}0$> _C40_// <$1\bar{1}0$]_C11b_ on the lamellar interface, due to the similarity in their crystal structures^11^. Furthermore, for the precipitated C11_b_-phase grains in the lamellar microstructure, three variant orientation relationships ideally exist. This is because of a slight distortion in the atomic arrangement on the common (0001)_C40_//{110)_C11b_ planes; the [110] axis in the C11_b_ structure and the [0001] axis in the C40 structure have two-fold and six-fold rotational symmetries, respectively. As a result, three C11_b_-phase variants - variant 1 (V1), variant 2 (V2), and variant 3 (V3) – produced from the C40 single crystals are denoted as follows:

1 $${\rm{V}}1:\quad {(0001)}_{C40}//{(110)}_{C{11}_{b}},\quad {[\bar{1}2\bar{1}0]}_{C40}//{[1\bar{1}0]}_{C{11}_{b}},\quad {[10\bar{1}0]}_{C40}//{[001]}_{C{11}_{b}}$$

2 $${\rm{V}}2:\quad {(0001)}_{C40}//{(110)}_{C{11}_{b}},\quad {[2\bar{1}\bar{1}0]}_{C40}//{[1\bar{1}0]}_{C{11}_{b}},\quad {[0\bar{1}10]}_{C40}//{[001]}_{C{11}_{b}}$$

3 $${\rm{V}}3:\quad {(0001)}_{C40}//{(110)}_{C{11}_{b}},\quad {[\bar{1}\bar{1}20]}_{C40}//{[1\bar{1}0]}_{C{11}_{b}},\quad {[\bar{1}100]}_{C40}//{[001]}_{C{11}_{b}}$$

These orientation relationships are hereinafter referred to as the variant orientation relationships. In addition, Supplementary schematics (Figure S2) are provided to explain these variant orientation relationships. It is also worth noting that in Fig. 1(a–d) the crystal orientation map was analyzed along a direction rotated towards [11 $\overline{2}$ 0] by 15° from the observed [10 $\overline{1}$ 0] direction, to distinguish the orientation colors of these three variant C11_b_-phase grains. Thus, red, pink, and purple grains in the map represent the C11_b_-V1, V2, and V3 grains, respectively.

A fine lamellar microstructure composed of C40/C11_b_ phases was developed in the nonadded ternary crystal, however, the existence of some coarse C11_b_-phase grains was also observed (Fig. 1a). These coarse C11_b_-phase grains do not show the aforementioned variant orientation relationships. A collapse of the fine lamellar microstructure was observed to result due to corresponding increases in the volume fraction of the coarse C11_b_-phase grains as the annealing period increases^11^. However, the characteristics of the microstructure were largely varied by Cr- and Ir-additions^22^. The Cr-addition could significantly improve the thermal instability of the lamellar microstructure observed in the nonadded crystal, via reducing the misfit strain on the lamellar interfaces. The specimen was entirely covered by the aligned lamellar grains with variant orientation relationships (Fig. 1b). On the other hand, the microstructure was significantly refined by the Ir-addition. However, the volume fraction of the C11_b_-phase grains that did not exhibit the variant orientation relationship was not significantly reduced relative to that as observed in the non-added crystal (Fig. 1c). Furthermore, a wholly different microstructure compared to others, named the “cross-lamellar microstructure”, was found to be developed by the coaddition of Cr and Ir, at the upper part of the FZ-grown crystal after annealing^26^ (Fig. 1d). Through the three-dimensional observation, it was confirmed that in addition to the fine lamellar microstructure, the cross-lamellar microstructure contained rod-like C11_b_-phase grains that extended along a direction perpendicular to the lamellar interface, as schematically shown in Fig. 1e. In this study, much of the effort is concentrated to clarify the effectiveness of these variations in microstructure for the improvement of the silicide crystal’s high-temperature creep strength, especially focusing on the “cross-lamellar microstructure”.

**Figure 2:**
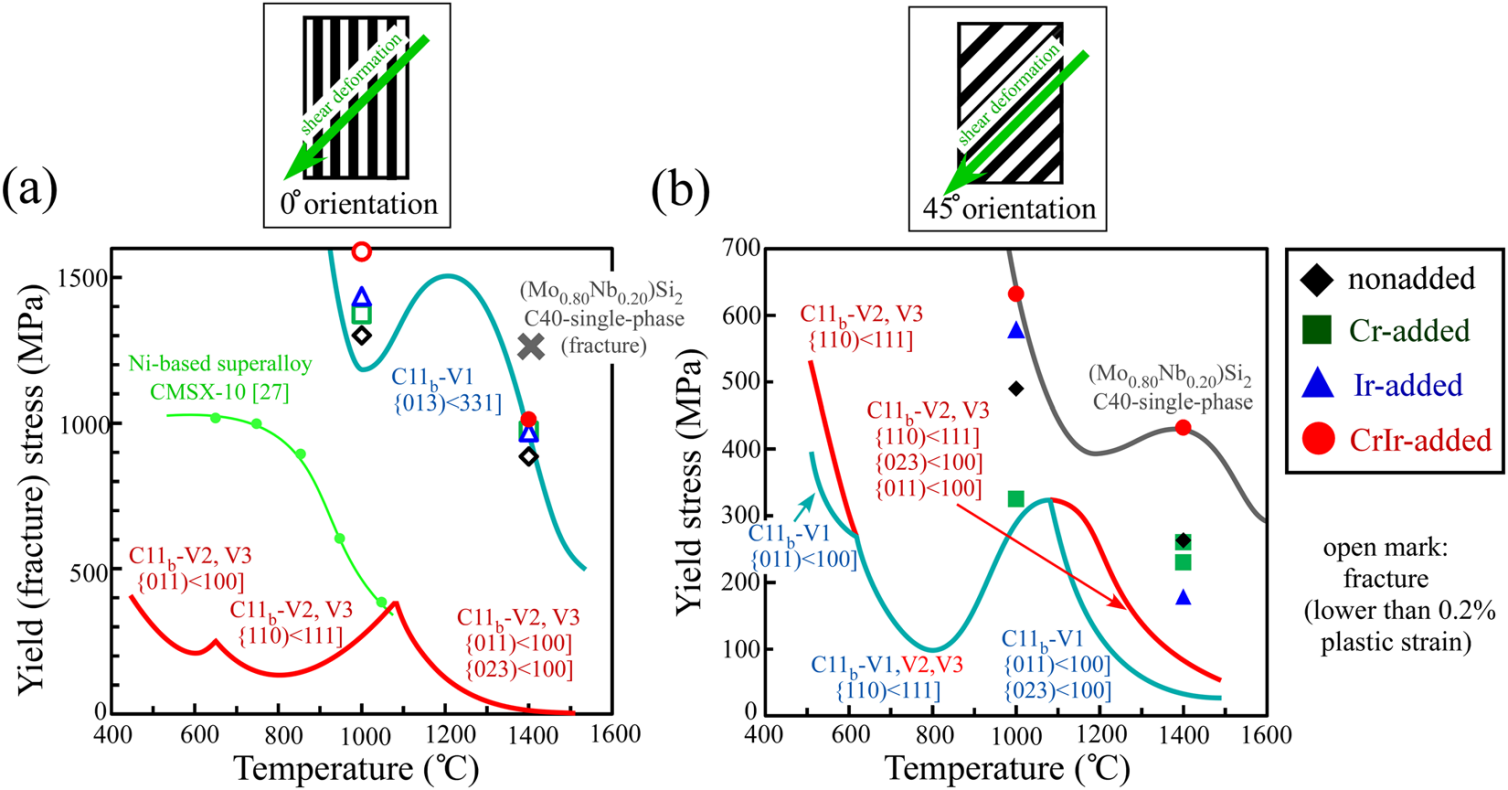
Variations in the yield (fracture) stress of the lamellar crystals. The yield (fracture) stresses were measured by compression tests at 1000 and 1400 °C in the (**a**) 0° and (**b**) 45° orientations. In the figures, the expected temperature dependencies of the yield stresses for the three C11_b_ variant grains and C40-matrix phase in the lamellar-structured crystals, according to the evaluation in the previous papers^5, 15, 24^, are also drawn for comparison. Open marks indicate that the specimen was fractured before reaching 0.2% plastic strain.

Prior to the creep tests, the high-temperature compressive behaviors of the crystals were examined. Figure 2 show the variations in the yield (fracture) stress of the (Mo_0.85_Nb_0.15_)Si_2_-based lamellar crystal as a function of the added element for deformation at 1000 and 1400 °C. The tests were conducted at two different loading orientations; one parallel to [10 $\overline{1}$ 0] and the other inclined by 45° towards [0001] in the [1 $\bar{2}$ 10] zone of the C40-matrix phase. Thus, the former is parallel to the lamellar interfaces, and the latter is inclined by 45° with respect to the lamellar interfaces. These loading orientations will hereinafter be referred to as the 0° and 45° orientations, respectively. The Schmid factors for the expected operative slip systems in the C11_b_ and C40 phases are listed in Table S1 in the Supplementary Information. In the graphs, the expected temperature dependencies of the yield stresses for the three C11_b_ variants and C40 phase, which are the predominant constituents in the lamellar crystal, were plotted according to the evaluation in the previous papers^5, 15, 24^. Note that the development of a lamellar microstructure means that in the 0°-oriented specimen, both of the C40 and C11_b_ phases must be plastically deformed for the specimen to be macroscopically deformed. In contrast, if the specimen is entirely composed of a perfect (plate-like) lamellar microstructure, then, in the ideal case for the 45°-oriented specimen, only the deformation of either the C40 or C11_b_ phase can induce macroscopic deformation, as represented schematically in the graphs.

In the 0° orientation, almost all specimens were observed to fracture before plastic deformation, and the fracture stress was much higher than that in the 45° orientation, irrespective of the added element. However, the details on the variations in the yield (fracture) stress due to the addition of the elements were different depending on the loading orientation and the added element. For deformation in the 0° orientation at 1000 °C, the addition of both Cr and Ir slightly increased the fracture stress, and the coaddition of Cr and Ir further increased the fracture stress. Furthermore, the addition of elements also increased the fracture stress at 1400 °C, but the variation in the extent of the increase in the fracture stress depending on the added element was small at 1400 °C. Note that plastic strain was only obtained for the CrIr-added crystal notwithstanding that the amount was as small as ~0.2%. The yield stress of the CrIr-added crystal was comparable to that expected in the C11_b_-V1 grain.

On the other hand, all specimens could be plastically deformed in the 45° orientation, and the yield stress showed significant variations with the added element, as shown in Fig. 2b. For deformation at 1000 °C, the refinement of the microstructure due to the addition of Ir increased the yield stress, and the effect was more significant for the CrIr-added crystal. In contrast, the addition of Cr drastically decreased the yield stress. At 1400 °C, the variation in the yield stress became small for the Cr-added and Ir-added crystals, and they exhibited comparable or slightly lower values to that of the nonadded crystal. On the other hand, the yield stress of the CrIr-added crystal was much higher than that of the nonadded crystal and it maintained a high value of ~430 MPa, even at 1400 °C, which is comparable to the yield (fracture) stress of the C40-phase single crystal.

Notably, the measured yield stresses in the 0° orientation was extremely higher than that of CMSX^®^-10, a third generation Ni-based superalloy^27^, as shown in Fig. 2a. Furthermore, even in the 45° orientation, which is the softer loading orientation, the yield stress is also higher than that of CMSX^®^-10 above 1000 °C in the CrIr-added crystals. This demonstrates that appropriate control of the crystal orientation in the lamellar crystals provides both superior strength at high-temperatures and deformability (workability), especially in the CrIr-added crystal.

**Figure 3:**
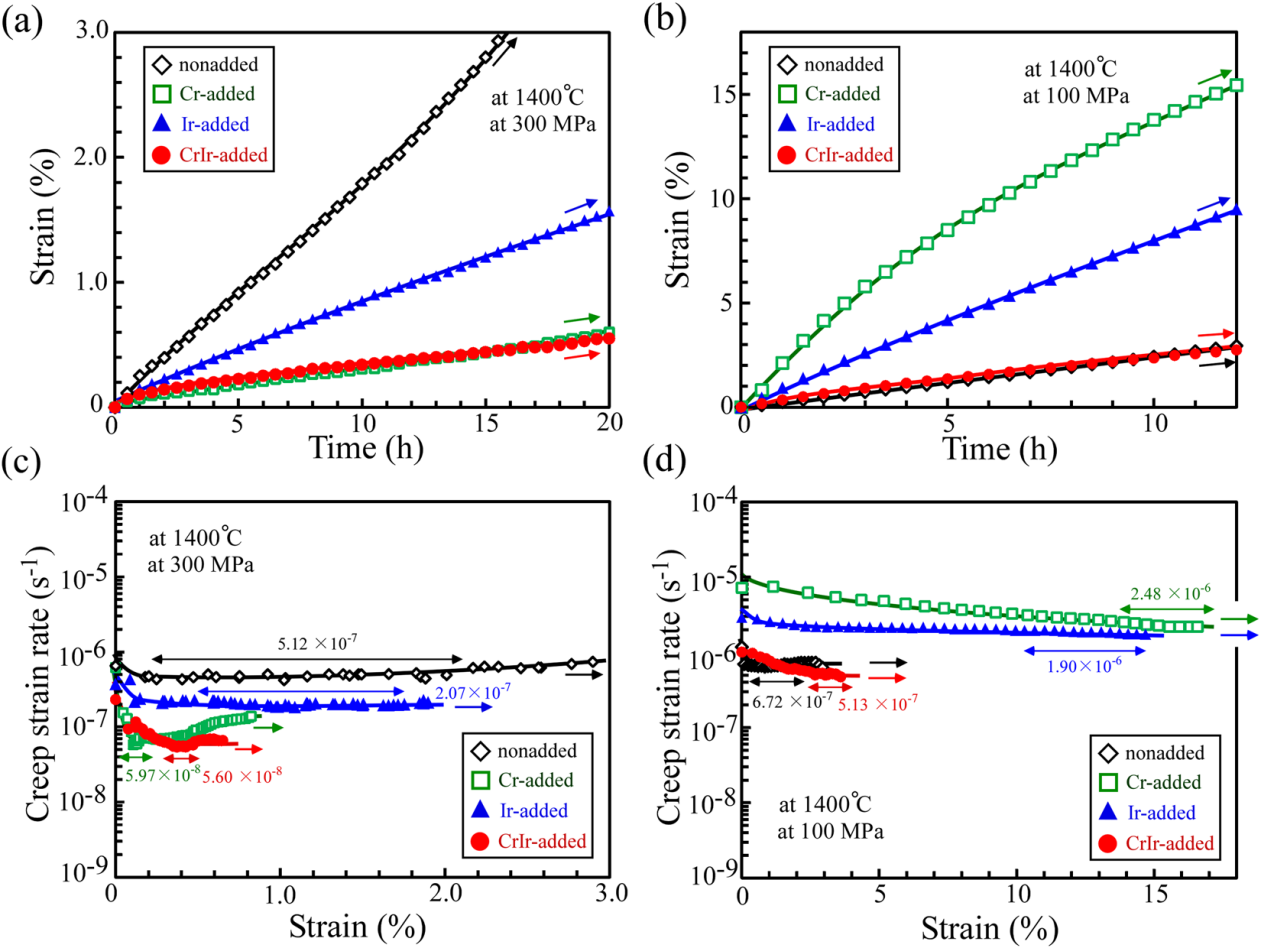
Compressive creep-deformation curves of the various (Mo_0.85_Nb_0.15_)Si_2_-based two-phase crystals at 1400 °C. (**a**) Creep strain versus time curves in the 0° orientation at 300 MPa and (**b**) in the 45° orientation at 100 MPa. (**c**,**d**) Corresponding creep strain rate versus strain curves for Figs. (**a**,**b**), respectively.

The variation in the mechanical properties of the added alloying element was more significantly observed from the high-temperature creep behavior. Figure 3(a,b) show the creep strain versus time curves for the various alloying-element-added crystals in the 0° and 45° orientations at 1400 °C. In addition, Fig. 3(c,d) show the corresponding creep strain rate versus strain curves to evaluate the minimum creep strain rate (MCR). In all of the crystals investigated, the creep strain was observed to be much larger in the 45° orientation than in the 0° orientation. However, the fine details of the variation in the creep behavior with the added alloying element were much different from one another.

In the 0° orientation, the addition of an alloying element improved the creep resistance, but the magnitude of the decrease in the creep strain rate was different depending on the added element. The magnitude of the decrease in the MCR followed the order of Ir-added crystal < Cr-added crystal ≤ CrIr-added crystal. In the Cr-added crystal and the CrIr-added crystal, the values of the MCR at 1400 °C and 400 MPa were almost comparable and/or slightly smaller than the smallest value of the MCR of a MoSi_2_ single crystal measured in the [001] loading orientation, which is the strongest orientation in the MoSi_2_^28^. In the CrIr-added crystal, the creep test was continued for ~500 h at 1400 °C and 150 MPa to examine its creep life. Notably, the specimen did not fracture, even after 500 h, and the creep strain was as small as ~1.0%, demonstrating its superior high-temperature creep resistance.

It must be mentioned that although the Cr-addition largely improved the creep resistance in the 0° orientation, it simultaneously reduced the creep resistance in the 45° orientation compared to that in the nonadded crystal, as shown in Fig. 3(a,b). This enhancement in the anisotropy of the creep deformation behavior is a serious problem for practical applications^24^. Similar behavior, i.e., an increase in the creep resistance in the 0° orientation accompanied by a decrease in the creep resistance in the 45° orientation was also observed for the Ir-added crystal. However, such a decrease in the creep resistance in the 45° orientation compared to that in the nonadded crystal was not observed in the CrIr-added crystal. The results demonstrate that the development of the cross-lamellar microstructure can first accomplish a large increase in the creep resistance in the 0° orientation without inducing a decrease in the creep resistance in the 45° orientation.

**Figure 4:**
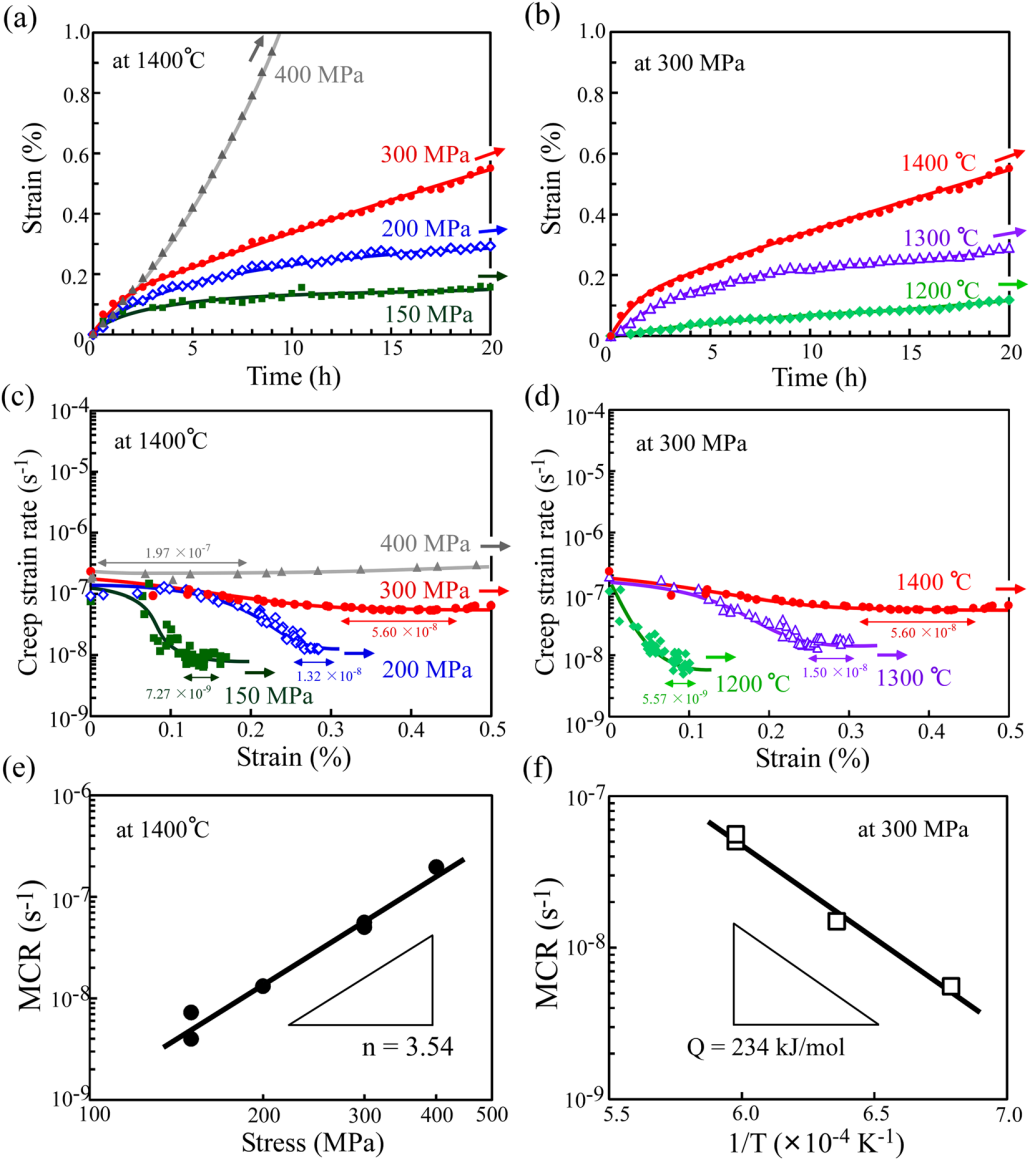
Compressive creep-deformation behavior of the CrIr-added crystals in the 0° orientation. (**a**) Creep strain versus time curves at 1400 °C and 150, 200, 300 and 400 MPa and (**b**) at 300 MPa and 1200, 1300, and 1400 °C. (**c**,**d**) Corresponding creep strain rate versus strain curves for Figs. (**a**,**b**), respectively. (**e**,**f**) Variations in the MCR in the 0° orientation (**e**) at 1400 °C as a function of the applied stress and (**f**) at 300 MPa as a function of the reciprocal of the temperature.

To clarify the origin of the improvement in the creep resistance and its controlling factors in the CrIr-added crystals with the cross-lamellar microstructure, the temperature and applied stress dependencies of the creep deformation behavior were examined at the 0° and 45° orientations. Firstly, Fig. 4(a,b) show the variations in creep strain versus time curves with the applied stress at 1400 °C and with the test temperature at the applied stress of 300 MPa, respectively, for the creep tests in the 0° orientation. In addition, Fig. 4(c,d) show the corresponding creep strain rate versus strain curves. For all specimens, after the relatively high-strain-rate region at approximately 0–3 h, a steady-state creep deformation region appeared. From the creep strain rate versus strain curves, the MCR was determined as depicted in the figures. The MCR increases when either the applied stress increases at a fixed temperature or the temperature increases at a fixed applied stress. Figure 4(e,f) show the relationships for the evaluated MCRs with the applied stress and the reciprocal of the temperature, respectively. From these plots, the stress exponent, n, and the apparent activation energy, Q, for creep in the 0° orientation were calculated to be 3.54 and 234 kJ/mol, respectively.

**Figure 5:**
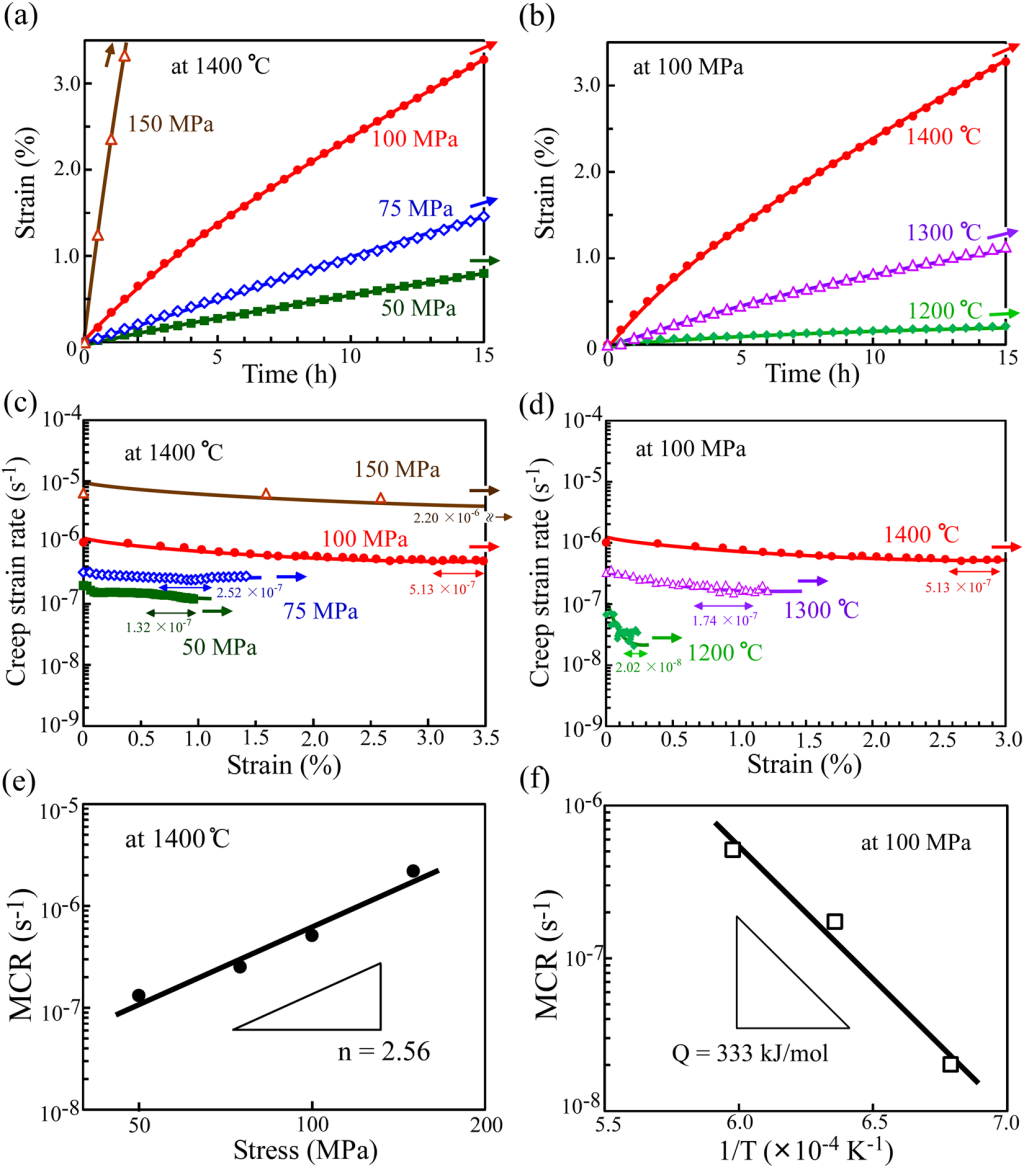
Compressive creep-deformation behavior of the CrIr-added crystals in the 45° orientation. (**a**) Creep strain versus time curves at 1400 °C and 50, 75, 100, and 150 MPa and (**b**) at 100 MPa and 1200, 1300, and 1400 °C. (**c**,**d**) Corresponding creep strain rate versus strain curves for Figs (**a**,**b**), respectively. (**e**,**f**) Variations in the MCR in the 45° orientation (**e**) at 1400 °C as a function of the applied stress and (**f**) at 100 MPa as a function of the reciprocal of the temperature.

Moreover, the variation in the creep deformation behavior with the applied stress and temperature for the CrIr-added crystals in the 45° orientation was examined, and the results are plotted in Fig. 5(a–f). Similarly, the MCR increases when either the applied stress increases at a fixed temperature or the temperature increases at a fixed applied stress. From these results, the values of n and Q for creep in the 45° orientation were calculated to be 2.56 and 333 kJ/mol, respectively.

**Figure 6:**
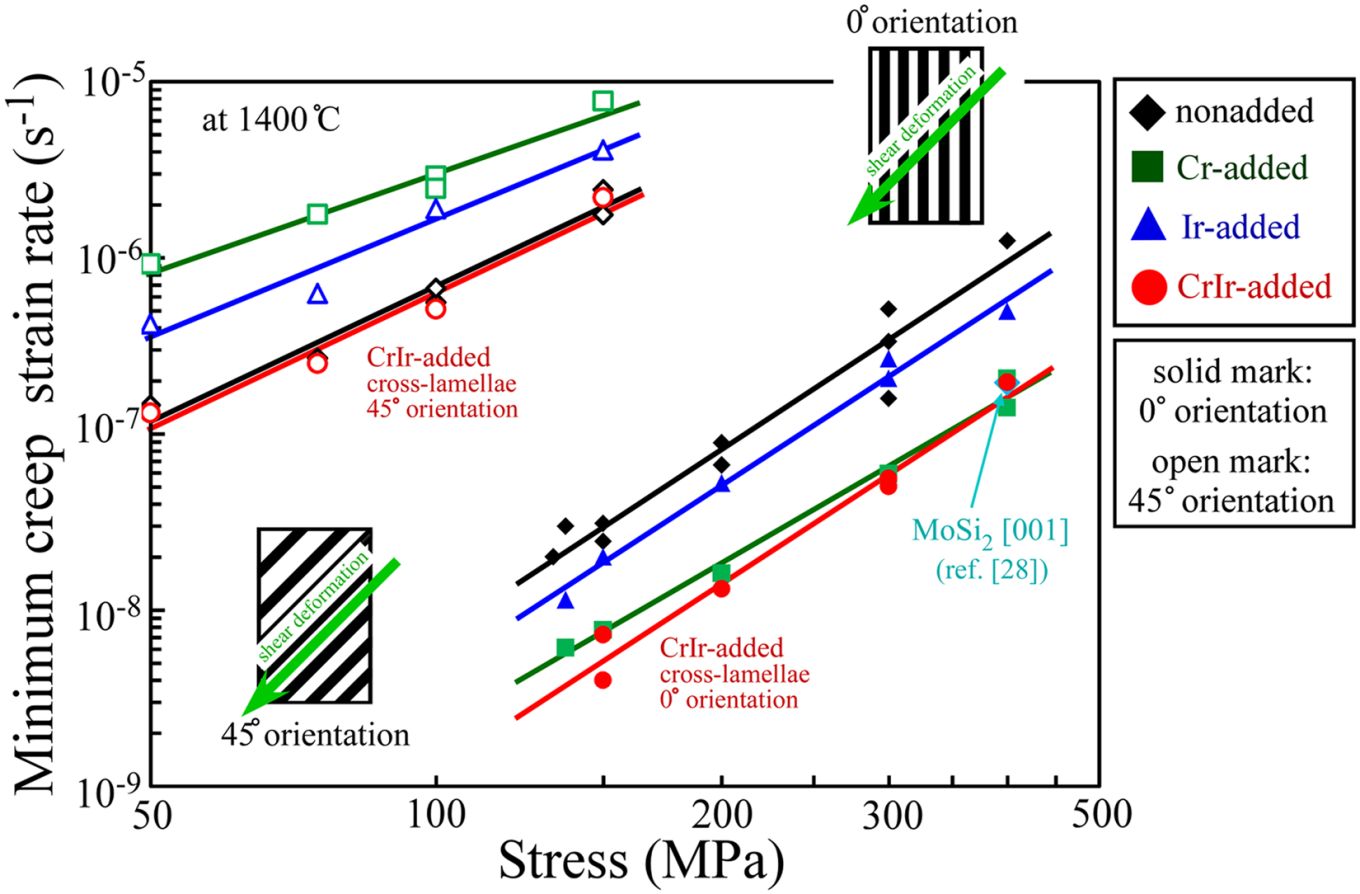
Summary of the creep strength in the lamellar crystals. Variations in the MCR with the applied stress and loading orientation in the creep tests at 1400 °C for the various (Mo_0.85_Nb_0.15_)Si_2_-based crystals investigated in this study are indicated.

To summarize the results, the relations between the MCR and the applied stress are plotted in Fig. 6, and the evaluated values of n are listed in Table 1 with the values of Q for nonadded and CrIr-added crystals. Since the variations in the creep deformation behavior with the applied stress in the Cr-added and Ir-added crystals were not known until now, the creep tests were also performed for those crystals herein, and the results are shown in Fig. 6 for comparison. The values of n and Q for the CrIr-added crystal with the cross-lamellar microstructure are relatively close to those measured for nonadded (Mo_0.85_Nb_0.15_)Si_2_ ternary crystals in both the 0° and 45° orientations. This suggests that the mechanisms controlling the creep deformation behavior themselves do not greatly vary for the CrIr-added crystals compared to those for the nonadded crystal. However, a large improvement of the creep resistance was achieved in the 0° orientation.

**Table 1:** The stress exponent n and activation energy Q for creep deformation for the various (Mo_0.85_Nb_0.15_)Si_2_-based two-phase crystals evaluated in this study.

	0° orientation		45° orientation	
	n	Q (kJ/mol)	n	Q (kJ/mol)
nonadded	3.48	284	2.50	381
Cr-added	3.13	—	1.88	—
Ir-added	3.53	—	2.19	—
CrIr-added	3.54	234	2.56	333

## Discussion

### Mechanism for the increase in the creep resistance in the 0° orientation

For deformation in the 0° orientation at 1400 °C, although the yield stress does not show marked differences, the creep deformation behavior was significantly varied by the addition of the alloying elements. The addition of alloying elements all decreased the MCR compared to that for the nonadded crystal, although the magnitude of the decrease was different in each crystal. The value of n in the 0° orientation was comparable for all crystals and about 3.1–3.5. These values are intermediate values between those measured for single crystals of C11_b_-MoSi_2_ (n = ~2.2–2.6^28, 29^) and C40-(Mo,Nb)Si_2_ (n = ~7.9^24^), suggesting that both phases contribute to the creep deformation of the lamellar crystal in the 0° orientation^30–32^, although it remains unclear why the Q-value at the 0° orientation (Q = ~284 kJ/mol in the ternary crystal) is lower than that at the 45° orientation (Q = ~381 kJ/mol) and is comparable to that of MoSi_2_, nevertheless that of C40-(Mo,Nb)Si_2_ (Q = ~1390 kJ/mol^24^) is much higher than that of MoSi_2_ (Q = ~251~372 kJ/mol^28, 29^). The mechanism governing the creep deformation behavior in the nonadded ternary crystal was previously discussed^24^ by focusing on the variations in the yield stress of the constituent C40 and C11_b_ phases described in Fig. 2. In the deformation at 0° orientation, the C40 phase is expected to act as an effective strengthening phase owing to the strong anisotropy of the deformation behavior. It has been reported that C40 phases primarily deform via basal slip^6, 7, 33^. However, the Schmid factor for basal slip in the C40 phase is negligible in the 0° orientation as shown in the supplementary Table S1. This is because the basal plane is parallel to the lamellar interface, which is, in turn, parallel to the loading axis. As a result, the C40 phase acts as an effective strengthening component in the 0° orientation. On the other hand, the C11_b_ phase is considered the ductile phase since five slip systems operate owing to its higher crystal symmetry^1, 4, 5^. However, the C11_b_-V1 grains with a [001] loading axis are also considered to act as a strengthening phase with a high yield stress, as shown in Fig. 2a. This is because only {013) < 331] slip, which exhibits a significant yield-stress anomaly^4, 5^, can be operative in the C11_b_-V1 grains, and the Schmid factors associated with the other slip systems are negligible along the [001] loading axis, as shown in Table S1.

**Figure 7:**
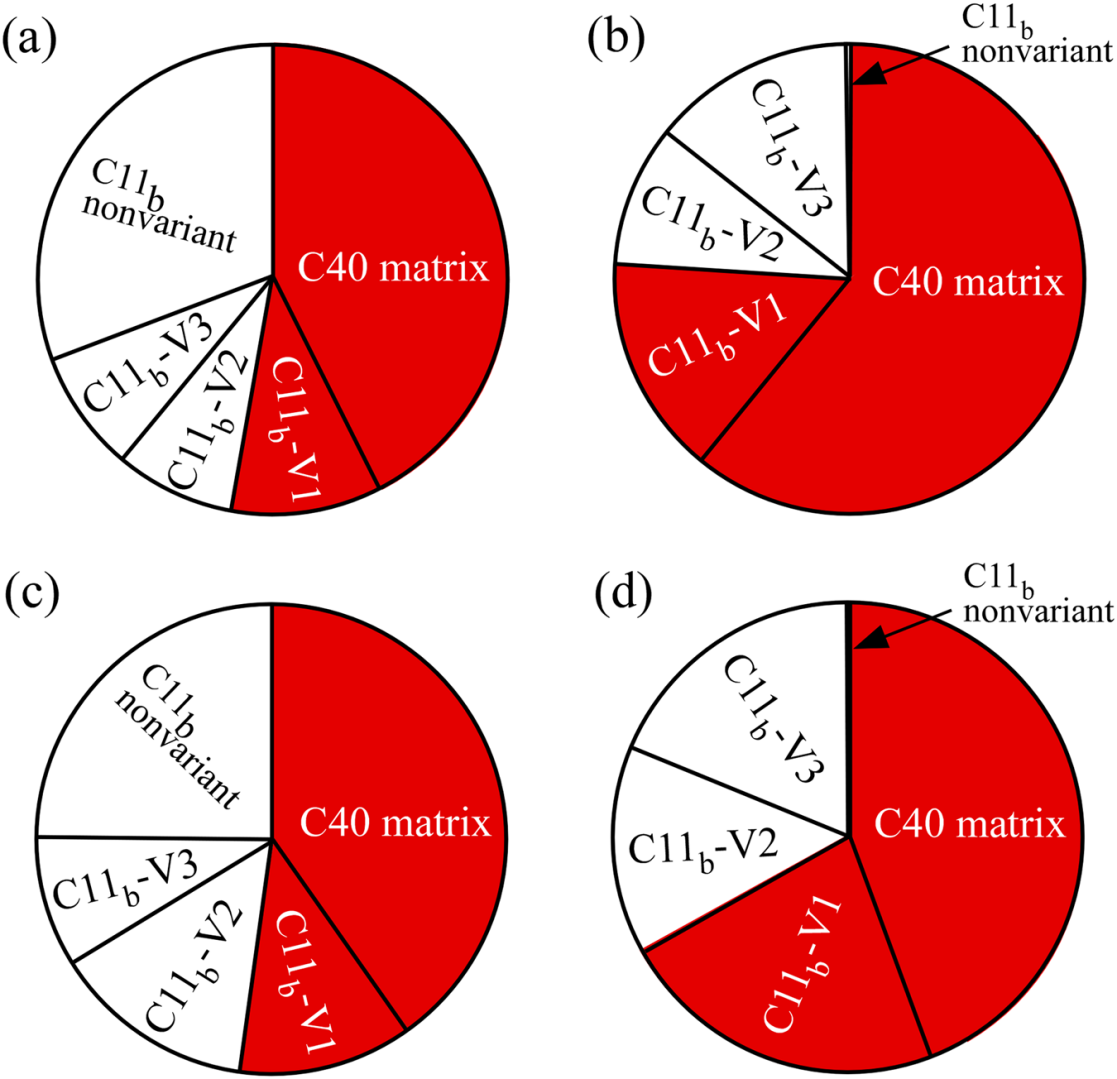
Pie charts showing the volume fractions of the constituent phases in the various (Mo_0.85_Nb_0.15_)Si_2_-based two-phase crystals. The values were estimated from the results of the SEM-EBSD analyses shown in Fig. 1. (**a**) nonadded crystal, (**b**) Cr-added crystal, (**c**) Ir-added crystal, and (**d**) CrIr-added crystal with cross-lamellar microstructure. The red segments represent the effective strengthening phases for creep deformation in the 0° orientation.

Based on these considerations, as one of the strengthening mechanisms by the development of cross-lamellar microstructure, the increase in the volume fraction of the effective strengthening component for creep deformation is considered. The volume fractions of the constituent phases in the (Mo_0.85_Nb_0.15_)Si_2_-based two-phase crystals investigated in this study (estimated from the results of the SEM-EBSD analyses shown in Fig. 1) is described in Fig. 7(a–d) using pie charts. In the pie charts, the red segments represent the strengthening phases of C40 and C11_b_-V1, as described above. As shown in Fig. 1d, the development of cross-lamellar microstructure in the CrIr-added crystal eliminated the coarse C11_b_-phase grains that do not exhibit the variant orientation relationship; thus, the volume fraction of C11_b_-V1 grains greatly increased. In addition, the volume fraction of the C40 phase also slightly increased compared to that of the nonadded ternary crystal. This is because of the increase in the phase stability of C40 by Cr addition^7^, as was more obviously detected in the Cr-added crystal (Fig. 7b). As a result, the volume fraction ratio of the strengthening phase in the CrIr-added crystal showed similar (significant) increases to that of the Cr-added crystal, as shown in Fig. 7d. This is one of the reasons why the creep resistance of the CrIr-added crystal significantly increased with the cross-lamellar microstructure.

On the other hand, in the Ir-added crystal, the volume fractions of the strengthening phases were close to that of the nonadded ternary crystal, since C11_b_-phase grains that do not exhibit the variant orientation relationship did not largely vanish as shown in Fig. 1c. However, its MCR moderately decreased compared to that of the nonadded crystal. This indicates that the refinement of the microstructure due to the addition of Ir also results in a modest decrease in the MCR of the silicide crystals. There are related reports describing reductions in the MCR due to the refinement of the microstructure for directionally solidified (DS) eutectic alloys such as MoSi_2_/Mo_5_Si_3_^34^ and Ni/Ni_3_Al/Cr_3_C_2_^35^, and in addition TiAl alloy^36^, which all exhibit aligned microstructures. As the detailed relationship between the creep strain rate and the lamellar spacing in such alloys, Mason and Van Aken^34^ reported that the creep strain rate, $\dot{{\rm{\varepsilon }}}$, of a DS MoSi_2_/Mo_5_Si_3_ crystal with an aligned microstructure can be described as follows:

4 $$\dot{{\rm{\varepsilon }}}={\rm{A}}\cdot {{\rm{\sigma }}}^{{\rm{n}}}\cdot {{\rm{\lambda }}}^{{\rm{m}}}\cdot \exp (-{\rm{Q}}/\text{RT})$$

where A is a rate constant, λ, the average lamellar spacing, m, the lamellar spacing exponent, R, the universal gas constant, and T, the absolute temperature. This assumption is based on the discussion given by Schmidt-Whitley^37^ for DS Al-CuAl_2_. In the model, the eutectic alloy is regarded as a fiber (or lamellar)-reinforced composite material, and the creep deformation is considered to be limited by the plastic deformation of the reinforcement, i.e., creeping reinforcements in a creeping matrix. Thus, the reduction in the lamellar spacing decreases the creep strain owing to the more effective transfer of the load by shearing from the matrix phase over the interface boundary to the stronger reinforcements. In addition, higher creep strength of the TiAl alloys with finer lamellar structures has been explained by enhanced strain hardening^36^. Based on these ideas, a factor related to the microstructural dependence of dislocation motion with changing microstructural dimensions, λ, was added to the Bailey–Norton creep model^34, 37^. The lamellar spacing exponent, m, in Equation (4) varies depending on the properties of the reinforcement, but it should be close to 1 for a perfectly aligned and continuous fiber (or plate)-reinforced composite^34, 37^. The value of m has not yet been experimentally determined for the C40/C11_b_ two-phase crystal, owing to the difficulty of the control of lamellar spacing in the crystal without changing the alloy composition. As a rough assumption here, assuming that the creep deformation mechanism is not varied in the nonadded and Ir-added crystals and m = 1, the ratio of the creep strain rates of the two crystals should correspond to the ratio of lamellar spacing in them, if the creep deformation obeys Equation (4). In the experimental results shown in Fig. 6, the ratio of average MCR at 300 MPa in them, i.e., ${\dot{\varepsilon }}_{{\rm{nonadded}}}$/${\dot{\varepsilon }}_{\text{Ir}-\text{added}}$, was estimated to be ~1.4. This value is relatively close to the ratio of the respective thicknesses of their fine lamellae^22^, i.e., ${\lambda }_{{\rm{nonadded}}}$/${\lambda }_{\text{Ir}-\text{added}}$ = ~1.8. This result implies that the refinement of the lamellae actually results in a decrease in the MCR of the C40/C11_b_ two-phase crystal via a similar mechanism, although further studies are required to confidently conclude this. Hence, the contribution of this grain refinement strengthening mechanism is expected in the CrIr-added crystal with the “fine” cross-lamellar microstructure.

In summary, the cross-lamellar microstructure provides the synergistic effects of the increase in the volume fraction of the strengthening components and their grain refinement strengthening, resulting in the drastic reduction in the creep strain rate of the CrIr-added crystal in the 0° orientation.

### Mechanism of the increase in the creep resistance in the 45° orientation

In the nonadded (Mo_0.85_Nb_0.15_)Si_2_ lamellar crystal, the creep strain in the 45° orientation was much larger than that in the 0° orientation. The same tendency was measured for all alloying-element-added crystals investigated in this study. However, the fine details of the creep deformation behavior were greatly different for each crystal. It is observed that the yield stress in the 45° orientation at 1400 °C was not so largely different, except for that of the CrIr-added crystal with the cross-lamellar microstructure (Fig. 2b), whereas the MCRs showed large variations with the added element (Fig. 3b). This suggests that different mechanisms may control the creep deformation behavior in each crystal in the 45° orientation.

In the creep deformation of the nonadded crystal in the 45° orientation, the strengthening effect by the C40 phase is weakened, and the deformation behavior of the C11_b_ phase is considered to predominantly govern the creep deformation behavior of the two-phase crystal. This conclusion is derived from the geometrical characteristics of the lamellar microstructure^24^. In the 45° orientation, ideally, only the deformation of the C11_b_ phase can carry the strain, which is different from the deformation in the 0° orientation, as schematically indicated in Fig. 2. Actually, the value of n for creep deformation in the 45° orientation was lower than that in the 0° orientation, and considerably close to that for a MoSi_2_ single crystal (n = ~2.6^29^), wherein the motion of < 100] dislocations carries the creep strain.

As describe in the previous section, the Cr-addition was effective to improve the creep strength in the 0° orientation. In the 45° orientation, however, the MCR showed a drastically increase as shown in Fig. 3b. This can be attributed to the development of the well-aligned lamellar microstructure shown in Fig. 1b. Due to the disappearance of the coarse C11_b_-phase grains, the obstacles hindering the motion of dislocations in the C11_b_-phase grains are lost in the lamellar microstructure, resulting in the drastic increase in the MCR. This enhancement in the anisotropy of the creep deformation behavior by Cr-addition is a serious problem that must be overcome for practical applications.

Note that the increase in MCR in the 45° orientation compared to that in nonadded crystal was also measured in the Ir-added crystal; nevertheless, much finer microstructure than that in the Cr-added crystal was developed in it. The reason for this has not been sufficiently clarified yet, but it is suspected that the refinement of the lamellar microstructure enhanced the occurrence of grain-boundary sliding in a localized region as an accommodation mode for creep deformation. The measured values of n that are close to 2 for the Ir-added crystal (n = 2.19) support this assumption. It must be mentioned, however, that the Cr-added crystal also showed a low n-value of 1.88, nevertheless the simple diffusion-controlled grain-boundary sliding was not expected to occur in it. Thus, further study focusing not only on the stress exponent but also on other factors is required to confidently conclude the creep deformation mechanism of them. As one of the possibilities, in addition to the diffusion-controlled grain-boundary sliding, the occurrence of localized deformation governed by the dislocation glide and climb in the vicinity of the C40/C11_b_ lamellar interface^38, 39^ is also suspected, since it may occur more easily and generate larger strain in the Cr-added and Ir-added crystals in which the lamellar interfaces are much flatter than that in the nonadded crystal owing to the disappearance of coarse C11_b_-phase grains.

However, the yield stress at 1400 °C for the CrIr-added crystal with the cross-lamellar microstructure is much higher than those for other crystals, and the MCR is maintained at a low value, which in comparable to that of the nonadded (Mo_0.85_Nb_0.15_)Si_2_ crystal. In other words, the CrIr-added crystal can greatly improve the creep resistance in the 0° orientation without degrading the creep resistance in the 45° orientation, unlike the Cr- or Ir-added crystals. Figure 1d shows that the development of cross-lamellar microstructure reduced the grain length of the C11_b_ phase along the lamellar interface in the CrIr-added crystal. Such microstructure refinement introduces many grain boundaries in the specimen. Ito *et al*.^4, 5^ reported that the number of operative slip systems is limited in C11_b_-MoSi_2_, and some of their critical resolved shear stresses (CRSSs), e.g., the CRSS for {013) slip, are extremely high. Thus, because of the difficulty in ensuring the strain continuity for deformation beyond the grain boundary, the grain boundaries act as effective obstacles for the motion of dislocations in the C11_b_ grains, including the < 100] dislocation, which governs the creep deformation of the nonadded crystal^24^. This leads to the increase in the creep strength of the CrIr-added crystal. In addition, as the advantage derived from the development of cross-lamellar microstructure, the existence of rod-like grains perpendicular to the lamellar interface can possibly prevent the occurrence of grain-boundary sliding and/or localized deformation along the lamellar interface, which were suspected to occur in the Ir-added crystal with the fine lamellar microstructure. Indeed, the value of n measured for the CrIr-added crystal was equal to 2.56 as shown in Table 1, which is much higher than that for the Ir-added crystal and is comparable to that for the nonadded crystal.

In conclusion, this study could achieve a large improvement in high-temperature creep strength, in addition to the room-temperature fracture toughness^26^, of a C40/C11_b_ two-phase lamellar crystal for the first time by the codoping of Cr and Ir. The cross-lamellar microstructure caused the MCR of the creep deformation to greatly decrease in the 0° orientation, where the applied stress was parallel to the lamellar interface. This was derived from the increase in the volume fraction of the effective strengthening phases of the C40 phases and variant-1-type C11_b_-phase grains in which the loading axis is parallel to [001]. Furthermore, the refinement of the microstructure was modestly effective for improving the creep resistance. In addition, the existence of rod-like C11_b_-phase grains could prevent the significant degradation in the creep strength for the loading orientation inclined from the direction parallel to the lamellar interface, which has been a serious problem for all the alloying-element-added C40/C11_b_ lamellar crystals until now. The development of a unique cross-lamellar microstructure was found to play a key role in the simultaneous improvements in the high-temperature creep strength and room-temperature fracture toughness.

## Method

Four types of mother alloys with chemical compositions of (Mo_0.85_Nb_0.15_)Si_2_, (Mo_0.85_Nb_0.15_)_0.97_Cr_0.03_Si_2_, (Mo_0.85_Nb_0.15_)_0.97_Ir_0.03_Si_2_, and (Mo_0.85_Nb_0.15_)_0.97_Cr_0.015_Ir_0.015_Si_2_, were prepared by arc melting high-purity Mo, Nb, Si, Cr, and Ir in an Ar atmosphere. The alloys are referred to as the nonadded ternary, Cr-added, Ir-added, and CrIr-added alloys, respectively in the manuscript. Single crystals with the C40 structure were grown using an optical FZ furnace (SCI-MDH-20020, Canon Machinery, Japan) at a growth rate of 2.5 mm/h in an Ar gas flow. The Laue X-ray diffraction method was used to confirm that the single crystals were composed of the C40 single phase and to determine their crystal orientations. The C40-phase single crystals were then annealed at 1400 °C for 168 h in an Ar atmosphere to develop the two-phase microstructure composed of the C40 and C11_b_ phases. The microstructure developed in the annealed crystals was examined by an OM (OLYMPUS BX51 M) and SEM (JEOL JSM-6500F). In addition, the crystal orientation relationship between the C40 and C11_b_ phases was examined by SEM-EBSD at a measured step distance of 0.3 μm.

To examine the high-temperature strength of the crystals, constant strain-rate compression tests and compressive creep tests were conducted. From the annealed C40/C11_b_ two-phase crystals, rectangular specimens with a cross section of 2.0 × 2.0 mm^2^ and a height of 5.0 mm for the compression test and specimens with a cross section of 2.4 × 2.4 mm^2^ and a height of 6 mm were cut for the creep tests by electrical discharge machining. The creep-test specimens had rectangular markers with a cross section of 0.4 × 0.8 mm^2^ and a length of 2.4 mm near both edges for precisely measuring the creep strain using a noncontact-type digital extensometer. The details of the experimental method for the creep test can be referred to in our previous paper^24^. Two loading axes were selected for the compression tests and creep tests: one parallel to [10 $\overline{1}$ 0] and the other inclined by 45° towards [0001] in the [1 $\bar{2}$ 10] zone of the C40-matrix phase. Thus, the former is parallel to the lamellar interfaces, and the latter is inclined by 45° with respect to the lamellar interfaces; these loading orientations are referred to as the 0° and 45° orientations, respectively in the manuscript. Compression tests were conducted at 1000 and 1400 °C at a nominal strain rate of 1.67 × 10^−4^ s^−1^, and creep tests were conducted at 1200, 1300, and 1400 °C under applied compressive stresses of 50–400 MPa in an Ar atmosphere. The creep tests were conducted for 12–20 h and then stopped before fracture for most of the specimens to evaluate the MCR in the steady-state and to examine the deformation microstructure. For some specimens, the creep tests continued up to ~500 h to examine the variation in the creep properties of the crystals with the test period.

## Electronic supplementary material

Supplementary information
