# Supplementary material for: Outstanding compressive creep strength in Cr/Ir-codoped (Mo0.85Nb0.15)Si2 crystals with the unique cross-lamellar microstructure
Source: Sci Rep. 2017 Jun 21;7:3936. doi: 10.1038/s41598-017-04163-0 (PMC5479845; doi:10.1038/s41598-017-04163-0)
Supplement: Supplementary file 1 — Supplementary information [file 41598_2017_4163_MOESM1_ESM.pdf]

SUPPLEMENTARY INFORMATION
for

Outstanding compressive creep strength in Cr/Ir-codoped $(Mo_{0.85}Nb_{0.15})Si_2$ crystals with the unique cross-lamellar microstructure

Koji Hagihara[1*], Takaaki Ikenishi[1], Haruka Araki[1], Takayoshi Nakano[2]

[1] Department of Adaptive Machine Systems, Graduate School of Engineering, Osaka University, 2-1 Yamadaoka, Suita, Osaka 565-0871, Japan

[2] Division of Materials and Manufacturing Science, Graduate School of Engineering, Osaka University, 2-1 Yamadaoka, Suita, Osaka 565-0871, Japan

* Corresponding author: hagihara@ams.eng.osaka-u.ac.jp

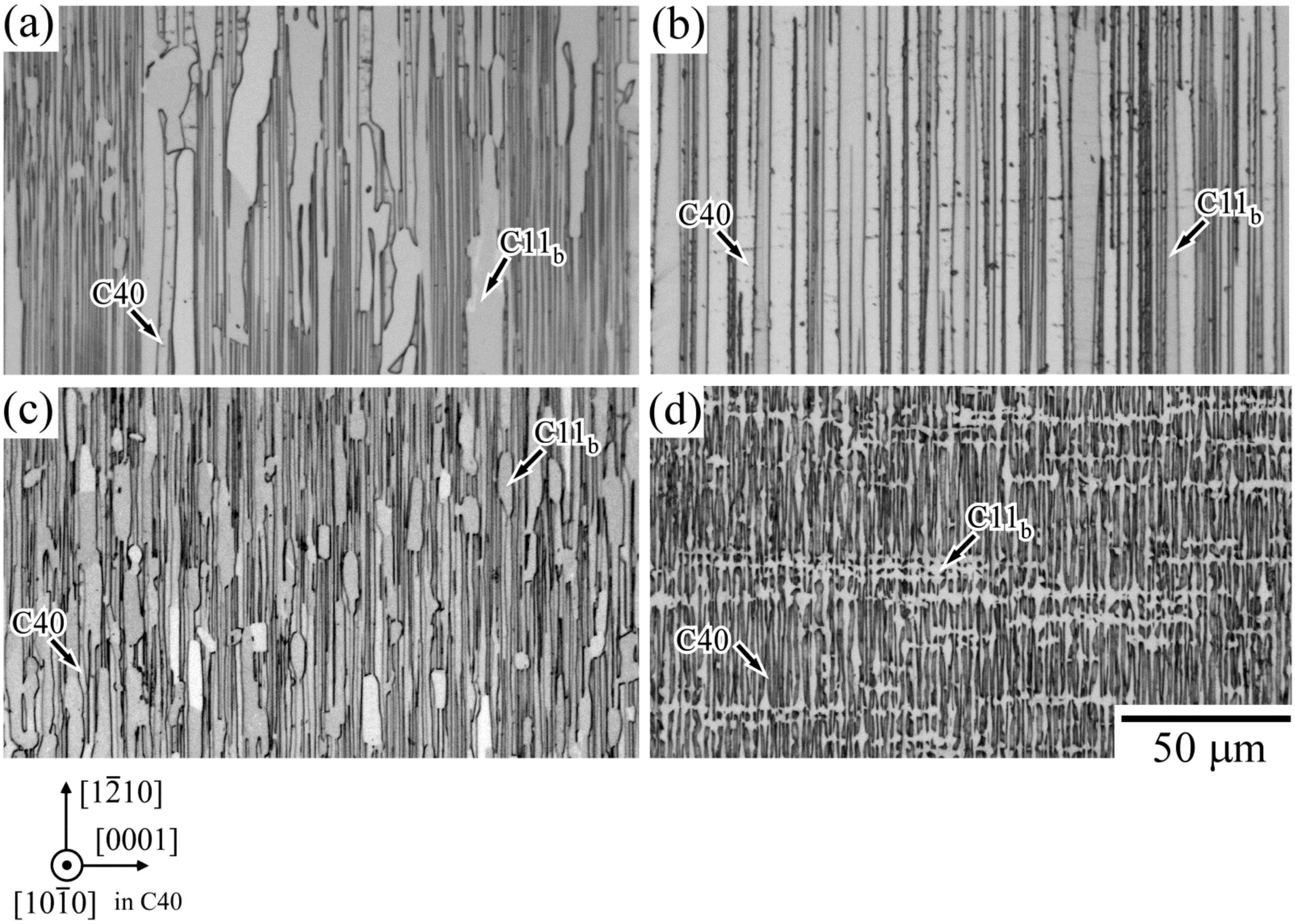

Supplementary Figure S1 | Microstructure developed in the annealed crystals.

Optical micrograph images showing the two-phase microstructures developed in the various FZ-grown crystals annealed at 1400 °C for 168 h. (a) Nonadded $(Mo_{0.85}Nb_{0.15})Si_2$ ternary crystal, (b) 1 at.% Cr-added crystal, (c) 1 at.% Ir-added crystal, (d) 0.5 at.% Cr and 0.5 at.% Ir-coadded crystal.

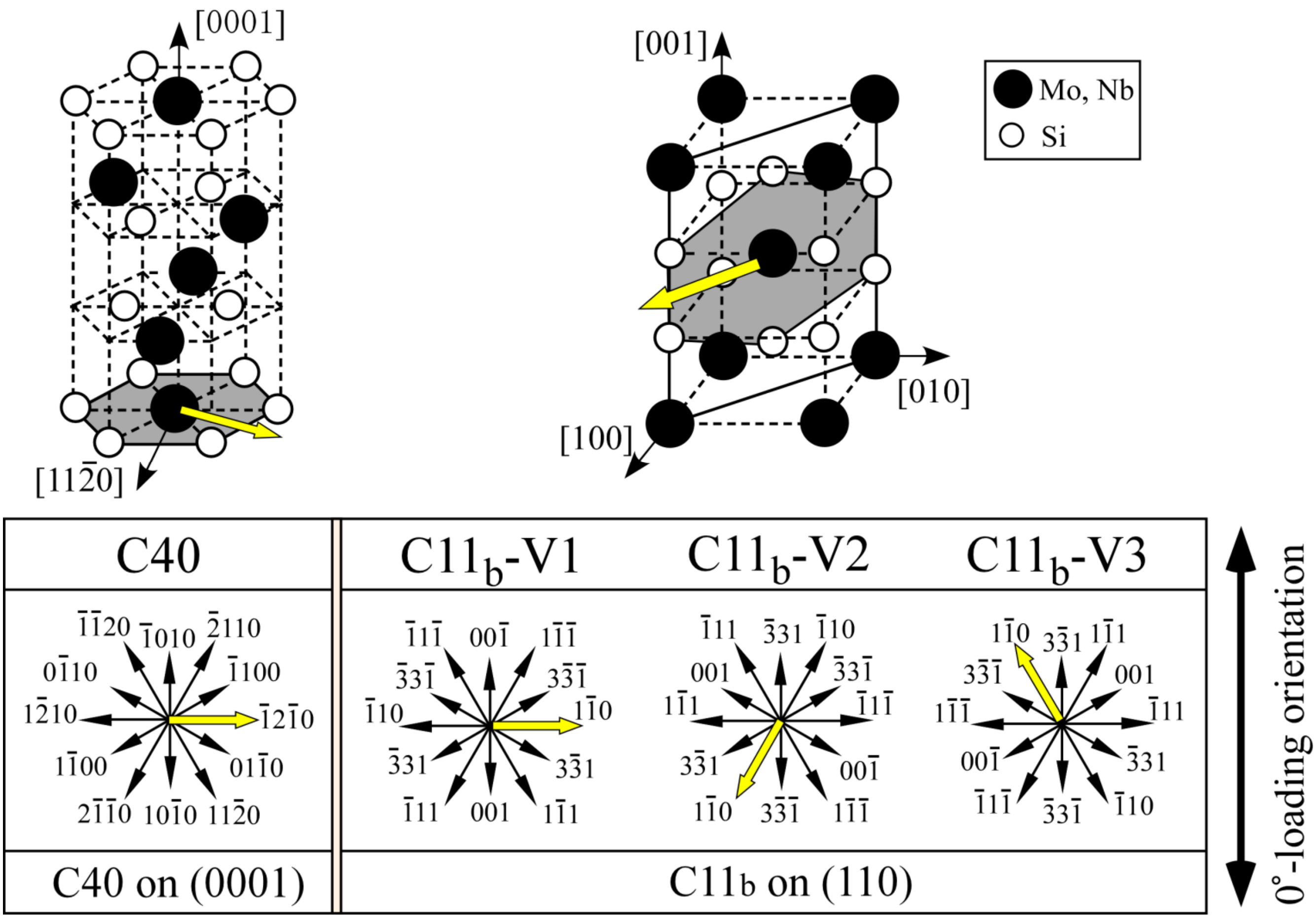

Supplementary Figure S2 | Orientation relationship in the lamellar crystals. Crystallographic orientation relationships between the C40-matrix phase on (0001) and the three variants (V1, V2, and V3) of the $C11_b$ phase on (110) in the lamellar-structured two-phase crystals[11] are schematically indicated with the crystal structures.

Supplementary Table S1 | Schmid factors for the possible operative slip systems.

The Schmid factors for the slip systems expected the operation in the C40-matrix phase and three $C11_b$ variants constituting the $(Mo_{0.85}Nb_{0.15})Si_2$-based two-phase lamellar crystals compressed in the 0° and 45° orientations, after reference [24] in the manuscript.

Slip system	0° orientation				45° orientation			
	Variant-type in $C11_b$			C40	Variant-type in $C11_b$			C40
	V1	V2	V3		V1	V2	V3	
Loading orientation	[001]	$[3\bar{3}\bar{1}]$	$[\bar{3}3\bar{1}]$	$[10\bar{1}0]$	[1.735 1.735 1]	$[6.465\ 0.465\ \bar{1}]$	$[0.465\ 6.465\ \bar{1}]$	$[1\ 0\ \bar{1}\ 1.228]$
$\{110)<1\bar{1}1]$	0	0.375	0.375	–	0.433	0.433	0.433	–
$\{010)<100]$	0	0.375	0.375	–	0.250	0.062	0.062	–
$\{011)<100]$	0	0.463	0.463	–	0.365	0.183	0.183	–
$\{023)<100]$	0	0.480	0.480	–	0.398	0.226	0.226	–
$\{013)<33\bar{1}]$	0.387	0.387	0.387	–	0.305	0.376	0.376	–
$(0001)<11\bar{2}0>$	–	–	–	0	–	–	–	0.433
